# Biodegradable polyelectrolyte/magnetite capsules for MR imaging and magnetic targeting of tumors

**DOI:** 10.7150/ntno.59458

**Published:** 2021-04-02

**Authors:** Yulia Svenskaya, Francesca Garello, Ekaterina Lengert, Anastasiia Kozlova, Roman Verkhovskii, Valeria Bitonto, Maria Rosaria Ruggiero, Sergey German, Dmitry Gorin, Enzo Terreno

**Affiliations:** 1 Remote Controlled Systems for Theranostics laboratory, Research and Educational Institute of Nanostructures and Biosystems, Saratov State University, 410012 Saratov, Russia.; 2 Molecular and Preclinical Imaging Centres, Department of Molecular Biotechnology and Health Sciences, University of Torino, 10126 Torino, Italy.; 3 Biomedical Photoacoustics Laboratory, Saratov State University, 410012 Saratov, Russia.; 4 Laboratory of Optics and Spectroscopy of Nanoobjects, Institute of Spectroscopy of the RAS, Troitsk 108840, Russia.; 5 Center of Photonics and Quantum Materials, Skolkovo Institute of Science and Technology, 143026 Moscow, Russia.

**Keywords:** magnetically-guided drug delivery systems, polyelectrolyte submicron capsules, magnetite nanoparticles, magnetic resonance imaging, targeted drug delivery.

## Abstract

**Rationale:** The tireless research for effective drug delivery approaches is prompted by poor target tissue penetration and limited selectivity against diseased cells. To overcome these issues, various nano- and micro-carriers have been developed so far, but some of them are characterized by slow degradation time, thus hampering repeated drug administrations. The aim of this study was to pursue a selective delivery of magnetic biodegradable polyelectrolyte capsules in a mouse breast cancer model, using an external magnetic field.

**Methods:** Four different kinds of magnetic polyelectrolyte capsules were fabricated via layer-by-layer assembly of biodegradable polymers on calcium carbonate templates. Magnetite nanoparticles were embedded either into the capsules' shell (sample S) or both into the shell and the inner volume of the capsules (samples C_n_S, where n is the number of nanoparticle loading cycles). Samples were first characterized in terms of their relaxometric and photosedimentometric properties. *In vitro* magnetic resonance imaging (MRI) experiments, carried out on RAW 264.7 cells, allowed the selection of two lead samples that proceeded for the *in vivo* testing on a mouse breast cancer model. In the set of *in vivo* experiments, an external magnet was applied for 1 hour following the intravenous injection of the capsules to improve their delivery to tumor, and MRI scans were acquired at different time points post administration.

**Results:** All samples were considered non-cytotoxic as they provided more than 76% viability of RAW 264.7 cells upon 2 h incubation. Sample S appeared to be the most efficient in terms of T_2_-MRI contrast, but the less sensitive to external magnet navigation, since no difference in MRI signal with and without the magnet was observed. On the other side, sample C_6_S was efficiently delivered to the tumor tissue, with a three-fold T_2_-MRI contrast enhancement upon the external magnet application. The effective magnetic targeting of C_6_S capsules was also confirmed by the reduction in T_2_-MRI contrast in spleen if compared with the untreated with magnet mice values, and the presence of dense and clustered iron aggregates in tumor histology sections even 48 h after the magnetic targeting.

**Conclusion:** The highlighted strategy of magnetic biodegradable polyelectrolyte capsules' design allows for the development of an efficient drug delivery system, which through an MRI-guided externally controlled navigation may lead to a significant improvement of the anticancer chemotherapy performance.

## Introduction

The search for effective drug delivery approaches is driven by the observation that many therapeutic agents failed due to their limited ability to reach the target tissue and their poor selectivity against diseased cells. Additionally, also the drug availability at the pathological site, determined by the release of the drug from its carrier, is a critical step. A wide variety of carriers have been investigated so far, including lipid-based delivery systems 1 and conjugates 2, polymeric 3, 4 and inorganic particles 5, 6, “host-guest” supramolecular adducts 7, 8, and naturally-occurring systems like lipoproteins 9, 10, proteins 11, 12, peptides 13, 14, viral capsids 15,16, bacteria 17, 18, cells 19,20 and extracellular vesicles/exosomes 21. Drugs can be released from the carrier spontaneously or through specific chemical (*e.g.* decreased pH, redox condition, enzymes) or physical triggering stimuli (*e.g.* ultrasound, heat, light, electric or magnetic fields) exploited either alone or in tandem 22-27. However, since the amount of drug that can be encapsulated is limited and only a small percent of carriers reaches the target tissue, the efficiency of drug delivery can be controversial. Specifically, the analysis of the drug delivery studies published in 2005-2015, has shown that only 0.7% (median) of the administered nanoparticle dose was delivered to a solid tumor 28. Such a poor efficiency is associated with the physiological barriers that a drug-carrying platform faces after its intravenous injection (for example, diffusion, flow and shear forces, aggregation, protein corona adsorption, phagocytic sequestration and renal clearance) 29, 30. In terms of cancer treatment, the additional limitations are attributed to the heterogeneity of tumor vasculature exhibiting the zones of both increased and sparse vascular density, hierarchical disorganization, serpentine structure and irregular branching 28. In addition to the limited therapeutic efficiency, such off-target tissue delivery raises toxicity concerns.

Various targeting strategies enabling a site-specific drug addressing have been developed to overcome the physiological barriers. Molecular systems of all-length scales, from small molecules to cells 31, 32, and external physical stimulations, like electric and magnetic fields, ultrasound, mechanical forces, light and temperature gradients, 33, 34 allow the control of the carrier navigation.

Magnetic targeting has been widely used owing to the ability of magnetic fields to penetrate most materials 34, 35. Moreover, magnetic fields can pass through the body safely, opening up the perspective of magnetic carrier delivery to deep tissues 36. Both static and varying field magnet systems have been extensively studied. However, clinical trials mainly utilize permanent magnets for magnetic targeting 36. A plenty of magnetic carrier types have been proposed and effectively applied for remotely controlled targeting purposes 37-40. Among them, magnetic polyelectrolyte multilayer capsules represent a unique delivery system allowing for remote navigation with magnetic field and *in-situ* release of encapsulated material, including triggered drug release in response to physical stimuli, such as light or ultrasound 41. Recently, the enhanced delivery effectiveness under external magnet application was demonstrated *in vitro* and *in vivo* for micron- 42, 43 and submicron-sized 44 polyelectrolyte multilayer constructs decorated with magnetite nanoparticles.

The monitoring of carrier delivery to a specific organ (or tumor in the case of anticancer therapy) is a separate challenging task. The doping of carriers by magnetite nanoparticles opens opportunities for their visualization and control of drug delivery/release steps by magnetic resonance imaging (MRI) *in vivo* 44,45. Moreover, the degradation of a carrier can be detected through the change in MRI contrast of adjacent tissues caused by the enhancement of distance between nanoparticles upon their liberation 46. This also provides an indirect detection of encapsulated substance release. Furthermore, initial contrast of polyelectrolyte capsules can be controlled by the change in their structure or composition 47. By this means, an appropriate structure of such capsules can provide the ideal balance between MRI contrast and magnetic navigation property while keeping the enhanced payload ability.

Furthermore, the use of drug delivery carriers doped with magnetic nanoparticles opens up the perspectives for local magnetic hyperthermia of adjacent cancer tissues. Since alternating magnetic fields enable the heating of magnetic nanoparticles, hyperthermia in addition to drug therapeutic effect can be granted if particles are localized within the tumor 48-50. Generation of local hyperthermia can be exploited also to induce the drug release from the delivered carriers 51-53 and to permeabilize the cell membranes, with the beneficial consequence of improving the drug diffusion in the lesion 54.

By this means, fabrication of magnetic drug-carrying containers that can provide the enhanced drug delivery efficiency together with the ability to monitor and prove this delivery is an important task in theranostics field, especially in anticancer theranostics. The aim of this study was to explore the magnetic biodegradable polyelectrolyte capsules, combining the externally controlled navigation and MRI visualization properties, *in vivo* in a breast cancer mouse model.

## Experimental Section

### Materials

Calcium chloride (CaCl_2_), sodium carbonate (Na_2_CO_3_), ethylene glycol (EG), poly-L-arginine (PA), dextran sulfate sodium salt (DS), sodium chloride (NaCl), ethylenediaminetetraacetic acid (EDTA), iron (III) chloride hexahydrate (FeCl_3_), iron (II) chloride tetrahydrate (FeCl_2_), potassium ferricyanide, hydrochloric acid (HCl) 37%, 10% neutral buffered formalin (NBF) and nuclear fast red (NFR) were purchased from Sigma-Aldrich (USA). Nitric acid (HNO_3_) 70 % for trace metal analysis was purchased from Thermo Scientific^TM^ (USA). Roswell Park Memorial Institute Medium (RPMI-1640), Dulbecco's Modified Eagle's Medium (DMEM), heat-inactivated fetal bovine serum (FBS), L-glutamine, trypsin/EDTA and penicillin-streptomycin mixture were purchased from Lonza (Belgium). Dulbecco's phosphate buffered saline (PBS), acridine orange (AO) and propidium iodide (PI) were purchased from Gibco (USA). Bradford Protein Assay was purchased from Bio-Rad Laboratories (USA). Milli-Q water was used in all experiments (Milli-Q Purification System, Millipore, Merck, USA).

### Magnetite synthesis

The synthesis of magnetite nanoparticles (MNPs) was carried out by chemical precipitation from di- and trivalent iron salts in the presence of a base. Initially, 1.3 g of FeCl_3_ and 0.48 g of FeCl_2_ were mixed and dissolved in 25 mL of water under room temperature, and 0.8 g of citric acid were dissolved in water of the same volume. Then, 170 mL of 0.1 M NaOH were placed into the reaction cell. To remove excessive oxygen, the nitrogen was bubbled through the reaction cell, as well as the solutions of iron salts and citric acid. The iron salts were injected into the reaction cell after its heating until 40 °C with active mixing. After this, the obtained suspension was left under active mixing and nitrogen pressure for 40 s resulting in black sediment formation of magnetite nanoparticles. 25 mL of citric acid were further added to the reaction cell under constant mixing and nitrogen pressure. Dialysis of magnetic hydrosol was carried out during 3 days in a 3 L vial under slow mixing (50 rpm).

The size and morphology of the obtained MNPs were characterized by means of transmission electron microscopy (TEM) using a FEGTEM microscope (JEOL, Akishima, Tokyo, Japan) operating at 200 kV. For this purpose, a drop of the nanoparticle suspension was deposited onto a lacey-carbon copper grid. Image analysis and statistics were performed using Image J free software.

The hydrodynamic radius of nanoparticles was measured by dynamic light scattering (DLS) using a Zetasizer Nano ZS instrument (Malvern Instruments Ltd, Malvern, UK).

### Preparation of the carriers

Magnetic polyelectrolyte capsules were fabricated via Layer-by-Layer (LbL) assembly of biodegradable polymers on calcium carbonate (CaCO_3_) templates 55. For this purpose, CaCO_3_ submicron particles were synthesized by precipitation from the mixture of CaCl_2_ and Na_2_CO_3_ water solutions at the EG presence 56. Equal volumes of 0.33 M salts were added each to EG in 2:10 volume ratio and rapidly stirred at 700 rpm for 3 hours. The synthesized CaCO_3_ particles were thoroughly washed twice with water and once with ethanol, afterwards they were dried for 30 min at 60°C.

Two different types of MNP-doped carriers were formed then: 1) containing MNPs in the polyelectrolyte shell - *sample S*; 2) containing MNPs in both, the shell and the inner volume of the capsules - *samples C_n_S*. Different C_n_S samples were obtained depending on the amount of incorporated magnetite - *samples C_1_S, C_2_S, C_6_S*.

To incorporate MNPs into the inner volume of the capsules, magnetite nanoparticles were loaded into pores of CaCO_3_ cores before the shell deposition via freezing-induced method 57. For this purpose, the weighted portion (40 mg) of dried CaCO_3_ particles was resuspended in 2 mL of 1.7 mg mL^-1^ MNPs water suspension and then kept in a freezing chamber at -20 °C for 2 h under slow constant mixing with a rotator TetraQuant R-1 (TetraQuant, Russia). After that, the samples were thawed at room temperature and centrifuged at 3800 g for 1 min to separate the particles pellet. Freezing/thawing cycles were repeated 1, 2 and 6 times (samples C_1_S, C_2_S, C_6_S, respectively).

In order to incorporate MNPs into the capsules' shell, magnetite nanoparticles were adsorbed from 0.3 mg mL^-1^ water suspension as a shell layer. The shells were formed by self-assembly method using biocompatible PA and DS polyelectrolytes dissolved in 0.15 M NaCl at 1 mg mL^-1^ concentration. For each shell layer formation, CaCO_3_ particles were resuspended in 1 mL of the adsorbing solution, and the deposition was carried out by continuous shaking for 10 minutes. At the end of each adsorption cycle, the suspension was centrifuged for 1 minute at 3800 g to sediment particles and to remove a supernatant liquid phase, and then particles were triply washed with water. After consequent deposition of six layers (PA/DS/PA/MNPs/PA/DS), CaCO_3_ templates were dissolved in 0.2 M EDTA, and the suspension of capsules was triply washed with water, centrifuged at 3800 g for 3 minutes, and resuspended in water. Thus, different hollow MNP-doped biocompatible polyelectrolyte submicron capsules were formed (samples S, C_1_S, C_2_S and C_6_S).

Pure six-layered polyelectrolyte submicron capsules made of PA and DS were prepared as a control. No incorporation of MNPs was made for this sample (neither into the shell nor into the inner volume of the capsules).

The morphology of the prepared polyelectrolyte capsules was characterized by scanning electron microscopy (SEM) using MIRA II LMU instrument (Tescan, Czech Republic) at an operating voltage of 20 kV. Size distribution of the carriers was investigated by a set of SEM images in order to obtain a minimum of 100 measurements per sample. Image analysis and statistics were performed using Image J free software. The average size was shown as “mean ± standard deviation”.

The Fe (III) content in magnetic polyelectrolyte capsules was determined relaxometrically through the glass vial test. For this purpose, aliquots of each sample were diluted 1:10 with HNO_3_ (70% w/w), transferred to glass ampoules and centrifuged for 3 min at 2000 rpm. The glass vials were sealed and placed at 120 °C overnight to mineralize the samples. Then, the longitudinal relaxation rate (R_1obs_) was measured at 21.5 MHz (0.5 T) and 25 °C using a Stelar Spinmaster spectrometer (Stelar srl, Italy). The temperature was controlled by a Stelar VTC-91 airflow heater (Stelar srl, Italy), equipped with a copper-constantan thermocouple (uncertainty was ± 0.1 °C). The millimolar concentration of Fe(III) was determined as following:


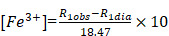
(1)

where *R_1dia_* (*R_1dia_* = 0.481 s^-1^) is the diamagnetic contribution in acidic conditions, while 18.47 mM^-1^ s^-1^ is the relaxivity (*r*_1_) of the Fe (III) aqua ion in acidic conditions at 21.5 MHz and 25 °C (a value determined by using standard FeCl_3_ solutions whose concentrations were measured by inductively coupled plasma mass spectrometry (ICP-MS), accuracy ± 0.1 %).

### Relaxivity measurements

Nuclear magnetic relaxation dispersion (NMRD) profiles of the control (pure) polyelectrolyte submicron capsules and MNP-doped ones (sample S) were recorded at 25 and 37 °C using a Stelar Fast Field Cycling FFC2000 NMR relaxometer (Stelar srl, Italy) operating at a magnetic field strength ranging from 0.01 to 20 MHz (2.4x10^-4^ to 0.47 T). The additional points were measured in the range of 20-70 MHz (0.47-1.65 T) using a tunable magnet (Stelar srl, Italy). The uncertainty of the measurements was lower than 5%.

The longitudinal relaxation rates of control (pure) polyelectrolyte capsule suspension in water measured at various proton Larmor frequencies were equal to water. Therefore, the polyelectrolyte shell did not affect the relaxivity of MNP-doped capsules and their relaxivity changes are caused only by the amount and disposition of magnetite nanoparticles in their structure. Thus, the normalized (to mM of Fe(III) ions) longitudinal relaxivity (r_1_) of magnetic submicron capsules was calculated according to the following equation:

r_1_
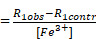
(2)

where *R_1contr_* is the diamagnetic contribution of pure water (*R_1contr_* = 0.38 s^-1^ at 21.5 MHz and 25 °C) 58.

Transverse relaxation rates (R_2obs_) of the obtained samples' suspensions were measured at 21.5 MHz (0.5 T) and 25 °C using a Stelar Spinmaster spectrometer (Stelar srl, Italy), the normalized (to mM of Fe(III) ions) transverse relaxivities (r_2_) were calculated then by subtracting the diamagnetic contribution of pure water from these values and dividing by the sample concentration.

Longitudinal and transverse relaxivity were also measured at 300 MHz (7 T), with a Bruker Avance 300 spectrometer (Bruker Biospin, Germany) equipped with a Micro 2.5 microimaging probe. The samples were transferred into glass capillaries and inserted into an agar phantom. T_1_ and T_2_ relaxation times were then measured. T_1_ measurement was performed with a Multi Slice Multi Echo (MSME) sequence varying the repetition time from 50 to 8000 ms (Echo Time (TE)/ Repetition Time (TR)/ Number of Averages (NAV) 3.74/50-8000/10, Matrix 128x128). T_2_ measurement of the samples was performed with a MSME sequence varying the echo time from 14 to 280 ms (TE/TR/NR 14-280/2000/1, Matrix 256x256).

### Sedimentation measurements

Mobility of the obtained capsules in an external non-uniform magnetic field was measured by photosedimentometry 47. The rate of capsules' sedimentation induced by a permanent neodymium magnet (340 mT) was measured using the previously described setup 57. The suspension of capsules in water was poured in the transparent cuvette, and 660 nm semiconductor laser beam was placed in parallel to the air/water interface. The magnet was applied to the cuvette's wall, so the magnetic field (326 mT at the cuvette's wall) was directed perpendicularly to the laser path. Thus, the time dependencies of the capsule suspension transparency under permanent magnet were obtained.

### Cellular experiments

#### Cells and Culture Conditions

Murine macrophage (RAW 264.7) and mammary adenocarcinoma (TS/A) cell lines were used in experiments. RAW 264.7 cells were purchased from American Type Culture Collection (ATCC LGC Standards, Sesto San Giovanni, Italy) and cultured in DMEM supplemented with 10% (v/v) of FBS, 2 mM L-glutamine, 100 U/mL penicillin and 100 μg/mL streptomycin at 37 °C in a humidified atmosphere with 5% CO_2_. TS/A cells, derived from a spontaneous BALB/c mammary tumor, were cultured in RPMI-1640 supplemented with 10% (v/v) of FBS, 2 mM L-glutamine, 100 U/mL penicillin and 100 μg/mL streptomycin at 37 °C in a humidified atmosphere with 5% CO_2_ 59.

#### *In vitro* MRI

MRI of macrophages after their incubation with the obtained magnetic polyelectrolyte capsules was carried out in order to check their ability to be labelled with the proposed system. To this purpose, 1x10^6^ RAW 264.7 cells were seeded into 60 mm Petri dishes. The day after the cells were incubated for 2 hours with MNP-doped capsules (samples S, C_1_S, C_2_S, C_6_S) at the concentration of 9x10^7^ capsules/mL in 2 mL of medium. The numbers of cells in suspensions, as well as the number of capsules in the samples, were counted using a hemocytometer. Incubation with medium was used as control. At the end of the incubation, cells were profusely washed to remove unbound particles, detached by scraping, resuspended in 10 mL of PBS and centrifuged twice at 1100 rpm for 5 min. Further, the cells were resuspended in 50 µL of PBS, transferred into glass capillaries and centrifuged at 700 rpm for 10 min to obtain a cell pellet. The capillaries were then inserted into an agar phantom and imaged at 300 MHz (7 T) with a Bruker Avance 300 spectrometer (Bruker Biospin, Germany) equipped with a Micro 2.5 microimaging probe. T_1_ weighted (T_1w_) Multi Slice Multi Echo MSME (Echo Time (TE)/ Repetition Time (TR)/ Number of Averages (NAV) 3.7/200/24, Matrix 128x128) and T_2w_ RARE (TE/Effective TE/TR/NAV/Rare Factor (RF) 3.9/31/4000/6/16, Matrix 128x128) sequences were acquired with an axial geometry (3 slices, slice thickness 0.7 mm).

The same experiment was performed for selected samples on breast cancer TS/A cells in order to simulate the process of capsules' uptake in tumor. For this purpose, 1x10^6^ TS/A cells were seeded into 60 mm Petri dishes. The day after the cells were incubated overnight (20 hours) or for 1 hour (the timeframe of magnet application planned for *in vivo* experiments) with MNP-doped capsules (samples C_1_S and C_6_S) at the concentration of 9x10^7^ capsules/mL in 2 mL of medium. At the end of the incubation, cells were also washed out from the unbound particles, detached with trypsin/EDTA, resuspended in 10 mL PBS and centrifuged twice at 1100 rpm for 5 min. The following procedure was the same as described above for RAW 264.7 cells.

Immediately after imaging, the cell pellets (both RAW 264.7 and TS/A) were recollected, resuspended in 200 µl of PBS and sonicated in ice with a Bandelin Sonopuls UW2070 probe sonicator (Bandelin, Germany) at 20 W for 30 s. The protein concentration in each sample was then measured spectrophotometrically using the Bradford protein assay and Jenway 6715 UV-Vis spectrophotometer (Jenway, USA). To quantify the amount of iron in each sample, cell extracts were digested with concentrated HNO_3_ (70% w/w, 1 mL) under microwave heating using a MicroSYNTH labstation (Milestone, Italy), recollected and analyzed by ICP-MS (Element-2, Thermo-Finnigan, Italy). The mean Fe(III) content per mg of protein was then calculated for each sample.

#### Cytotoxicity

Cytotoxicity measurements were performed for RAW 264.7 cell line to estimate the influence of the obtained MNP-doped capsules on the healthy cells. For the quantification of live and necrotic cells after their incubation with capsules' suspension, 2x10^5^ cells were placed on the 60-mm Petri dish and incubated overnight. Then a different number of MNP-doped carriers (9, 45, and 90x10^6^ capsules) in 2 mL of growth medium was added to cells and incubated for 2 hours to simulate the conditions of capsule injection in the bloodstream. The largest number of capsules (90x10^6^) added to cells was chosen the same as injected further to an average mouse (weighing 20 g) *in vivo*. The positive control contained RAW 264.7 cells without any additional treatment, the negative control was prepared by adding 30% v/v of ethanol to the macrophages. The cells of all the groups were detached then and stained with 0.125 μg/mL Acridine orange (AO) and 15 μg/mL Propidium iodide (PI) for 15 min. Finally, the macrophages were triple washed with PBS and measured using an imaging flow cytometer Amnis Mk II (Luminex, USA).

The analysis of the obtained data was performed using the IDEAS software (Luminex, USA). PI-only positive cells were recognized as necrotic ones. AO-positive and double-positive cells were determined as viable cells, where double-positive staining was caused by the adsorption of nucleic acids from the surrounded dead cells and their debris on the surface of the viable ones. The data on RAW 264.7 cell viability after their incubation with MNP-doped capsules were represented as “mean ± standard deviations” (n = 5). The one-way analysis of variance (ANOVA) was used to determine the statistical significance of differences in the obtained values. Calculations were made with Microsoft Excel software.

### *In vivo* experiments

#### Tumor animal model

The animal study was approved by the Italian Ministry of Health, the following procedures were in accordance with institutional guidelines and ensured the humane care of the animals. The mice (aged 8 weeks and weighting 18-22 g) were obtained from the animal facility at the Molecular Biotechnology Center of the University of Turin. A xenograft breast tumor mouse model was prepared by subcutaneous injection of 2.5x10^5^ adenocarcinoma TS/A tumor cells into the flank of BALB/c female mice. Ten days after tumor implantation, when tumor size reached a diameter of 3-6 mm, the animals were enrolled in the *in vivo* imaging studies.

#### MRI experiments

MRI *in vivo* experiments were performed at 40 MHz (1 T) with an Aspect M2 High-Performance MRI System (Aspect Magnet Technologies Ltd, Israel) consisting of a NdFeB magnet, equipped with a solenoid Tx/Tr coil of 35 mm inner diameter. This system was equipped with fast gradient coils (gradient strength of 450 mT m^-1^ at 60 A, ramp time of 250 μs at 160 V) with a field homogeneity of 0.2-0.5 gauss. Before MRI experiments, the animals were anesthetized by intramuscular injection of tiletamine-zolazepam (Zoletil 100; Virbac, Milan, Italy) 20 mg kg^-1^ and xylazine (Rompun; Bayer, Milan, Italy) 5 mg kg^-1^.

The suspension of magnetic polyelectrolyte capsules was diluted in HEPES/NaCl buffer (to obtain a suspension 280 mOsm, pH 7.4) and injected into the tail vein. The mice underwent MRI before and at different time points (1, 4.5, 24 and 48 hours) after the capsule injection. A standard T_2_ Fast Spin Echo sequence was used with the following parameters: TR/TE/NEX 2500/49/4, resolution of 250 μm, slice thickness of 1.5 mm.

Image analysis and statistics were performed using Image J free software. The mean signal intensity (*SI*) values were calculated on a region of interest (ROI). The measured *SI* was normalized to a water-containing reference tube to take into account the differences in the absolute signal intensity values (*SI_n_*, where n means “normalized”) among different images obtained after mouse repositioning in the MRI scanners. The normalization was carried out by dividing the *SI* values of the ROI drawn on the organ of interest to the *SI* values of the ROI drawn inside the reference tube (*SI_ref_*):


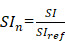
(3)

The mean percent of signal change (*SC*) was calculated according to the following equation:


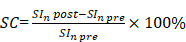
(4)

where *SI_n pre_ -* the mean signal intensity of the organ of interest before the capsule injection, *SI_ post_*- the mean signal intensity of the organ of interest after the capsule injection.

The data were calculated from six independent experiments, unless otherwise stated, and expressed as the “mean ± SE”. ANOVA and the *t*-test were performed to determine the statistical significance of differences in SE in tumor between different time points for the same experimental group (*p* < 0.05) and between different experimental groups (*p* < 0.01).

#### Magnetic targeting

**Figure 1:**
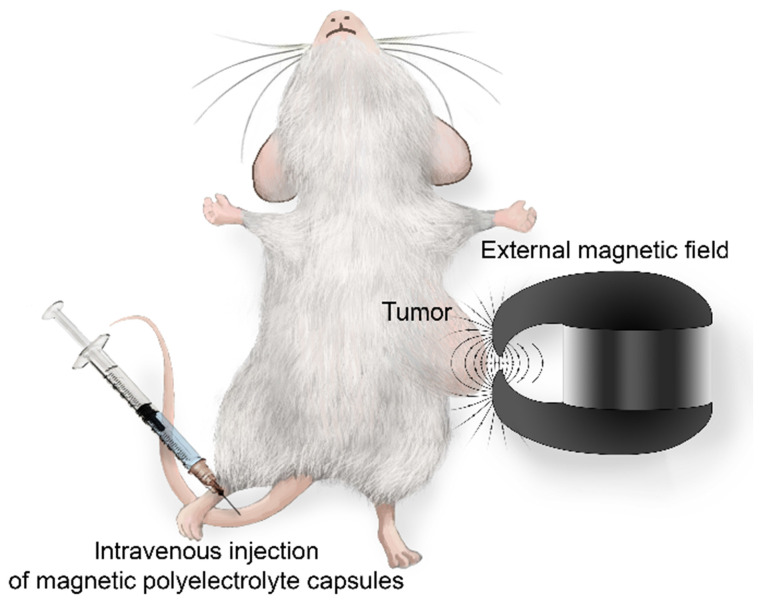
Schematic illustration of the magnetic targeting for MNP-doped capsules.

Active magnetic addressing of MNP-doped capsules was performed by the application of a permanent magnet with a concentrator (0.5 T) to the tumor. First, the magnet was placed on the tumor, and then the contrast agent was injected into the tail vein (Figure 1). The magnet was kept applied to the tumor for 1 hour.

#### Histology

For *ex-vivo* histological studies, tumor, liver and spleen were excised at different time points post injection and fixed overnight in a 10% NBF, dehydrated and embedded into paraffin. Then tissue slices (5 µm of thickness) were cut with a microtome. The presence and localization of iron was investigated by means of Perls' Prussian blue staining. Briefly, sections were hydrated, covered with equal parts mixture of 5% potassium ferrocyanide and 5% HCl for 1 hour, washed in distilled water and counterstained with NFR for 10 minutes. Finally, sections were dehydrated, cleared, mounted and examined under an Olympus BX14 microscope (Olympus, USA).

## Results and Discussion

### *In vitro* characterization of magnetic polyelectrolyte capsules

Layer-by-layer (LbL) assembled polyelectrolyte capsules represent a unique tool to fabricate micron- and submicron-sized theranostic systems which are capable of targeted delivery and controlled *in-situ* release 41, 60-64. So far, effective encapsulation of different drugs, including anticancer 65-68, RNA/DNA 69-71, growth factors 72, 73, antigens 74-76, and other bioactive substances 77-81 was demonstrated by means of polyelectrolyte capsules. Various multifunctional imaging agents were successfully fabricated using LbL technique as well 82-84. Embedding of magnetite nanoparticles into the structure of polyelectrolyte capsules opens up the perspective for their application in MRI 44, 85-88 as well as for improving drug delivery and controlled drug release 27, 41, 42, 44.

Considering this, magnetic nanoparticle (MNP)-doped polyelectrolyte capsules of different structure were formed and tested for capability of their delivery to tumor and MRI-visualization.

To start with, the colloid of MNPs was synthesized by the chemical precipitation method in an inert atmosphere 89, 90. A TEM image of the obtained nanoparticles is presented in Figure SM1 (Supplementary Material, SM). The image demonstrates a spherical shape of MNPs with an average size of 5.0 ± 0.9 nm. DLS measurements show a particle hydrodynamic diameter of 8.8 ± 2.0 nm (Figure SM1 in Supplementary Material).

**Figure 2:**
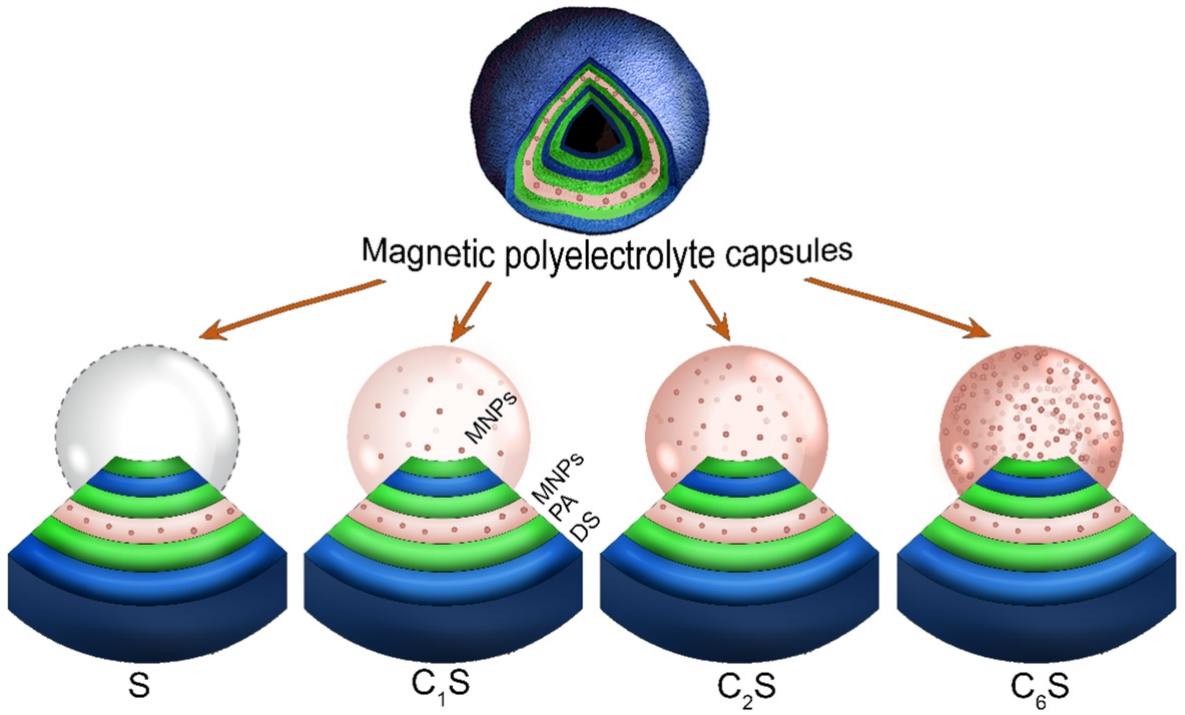
Scheme representing the structure of the obtained magnetic polyelectrolyte capsules: sample S containing magnetic nanoparticles (MNPs) in the capsules' shell and samples C_n_S containing MNPs in both, the shell and the inner volume of the capsules (n is the number of MNPs loading cycles). All the sample types contain poly-arginine (PA) and dextran sulfate (DS) polymers in their shell.

Magnetic polyelectrolyte capsules were then formed using this MNP suspension. Different capsule structures were formulated in order to determine a balanced carrier composition in terms of magnetic and MRI contrast properties. To accomplish this, MNPs were embedded either into the capsules' shell (sample S) or into both, the shell and the inner volume of the capsules (samples CnS, where n is the number of MNPs loading cycles). The scheme representing the structure of the obtained capsules is provided in Figure 2. The iron content in the obtained capsules (determined as Fe(III)) depending on their structure is presented in Table 1.

**Figure 3:**
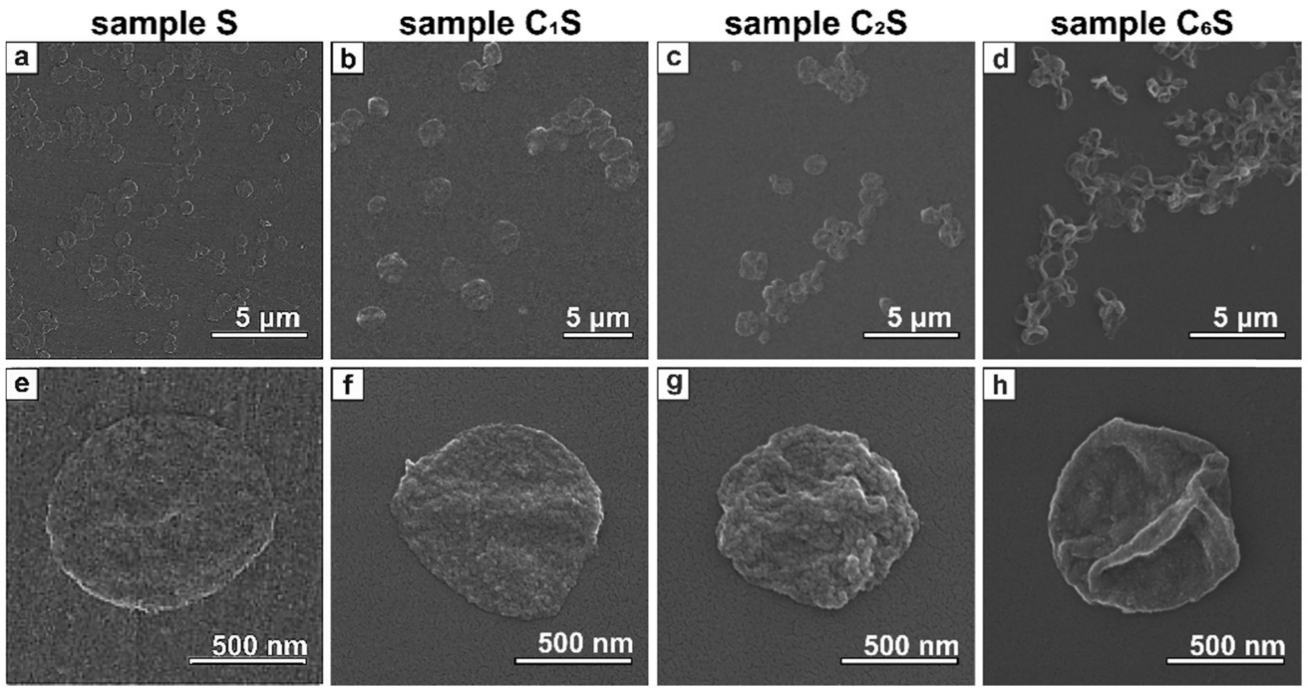
SEM images of magnetic polyelectrolyte capsules: (a, e) - sample S*;* (b, f) - sample C_1_S; (c, g) - sample C_2_S; (d, h) - sample C_6_S performed at different magnifications.

Biodegradability is a crucial property of drug delivery systems. For micron- and submicron-sized capsule formulation, the biodegradable polymers, either synthetic or natural, are often used since they are capable of being split into biocompatible products by chemical or enzyme-catalyzed hydrolysis 91. Poly-L-arginine (PA) and dextran sulfate (DS) have been widely employed to design polyelectrolyte capsules that were found to be biocompatible and degradable both *in vitro* and *in vivo* 92, 93. Thus, here the MNP-doped capsules were fabricated using these polymers by LbL technique. SEM images of the obtained carriers are presented in Figure 3. The average size of the capsules was 0.8 ± 0.1 μm.

The SEM images clearly demonstrated that the morphology of the capsules' surface differed depending on the amount of incorporated magnetite. In particular, sample S containing MNPs only in polyelectrolyte shell represented thin submicron capsules with quite smooth surface (Figure 3 a, e). Embedding of MNPs into the inner volume of the capsules resulted in increased wall thickness (Figure 3 b-d and f-h). Moreover, the higher magnetite content was in capsules, the rougher and thicker they appeared.

According to our previous data 46, the concentration of MNPs adsorbed at the shell defines MRI contrast properties of polyelectrolyte capsules. Specifically, the MR contrast for both T_1_ and T_2_ was shown to depend on the distance between magnetite nanoparticles in the capsules' shell. With the larger distance between MNPs in the shell layer, relaxation rate increases resulting in MR signal enhancement. In the current study, the amount of magnetite in a shell was chosen to provide the best MRI contrast 46. However, the polyelectrolyte carriers containing MNPs only in their shell possessed low sensitivity to magnetic field due to insufficient magnetite concentration 47. Meanwhile, the embedding of higher amount of MNPs into the structure of polyelectrolyte capsules can open up the possibilities for their navigation with external magnetic field 42, 44, 94, 95.

**Figure 4:**
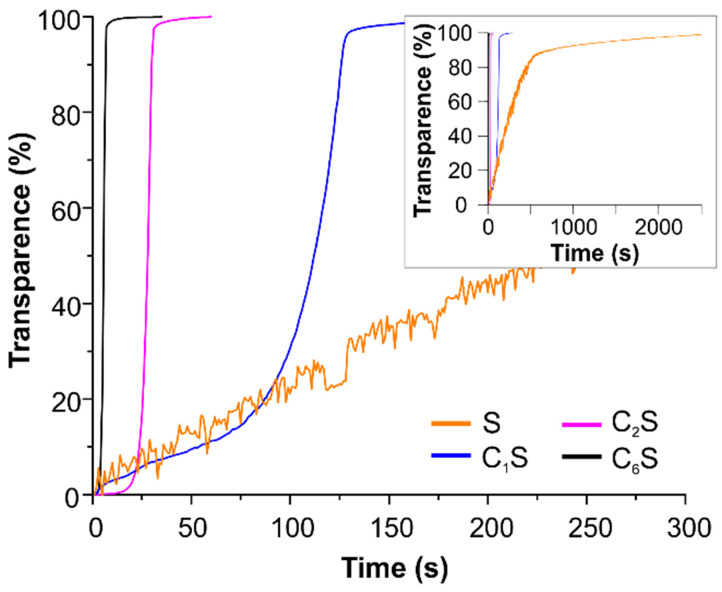
Photosedimentometry of magnetic polyelectrolyte capsules S, C_1_S, C_2_S and C_6_S dispersed in water during the time represented as time dependence of the suspension transparence in an external magnetic field.

The opportunity to improve the magnetic navigation property of polyelectrolyte capsules by the incorporation of MNPs into their inner volume was studied here. The effect of changing the amount of incorporated magnetite on the mobility of the obtained capsules in an external magnetic field was measured by means of photosedimentometry. The results are represented in Figure 4. The graphs demonstrate the change in transmission of the capsule suspensions under the application of a magnet depending on the capsules' structure. The photographs of the capsule suspensions in cuvette before and after the magnet application are presented in Figure SM2 in Supplementary Material.

According to the obtained data, the sample C_6_S was the most sensitive to an external non-uniform magnetic field. However, it possessed the lowest r_1_ and r_2_ relaxivities at 21.5 MHz (0.5 T) (Table 1). In contrast, the sample S without magnetite in the inner volume revealed as the best T_2_ contrast agent among all studied carriers with an r_2_/r_1_ ratio of 4.7. The T_1_-NMRD profile for this sample (at 25 and 37°C) is shown in Figure SM3, and it is characterized by a broad hump centered at around 10 MHz. It should be noted that the obtained profile appears very similar to Endorem™, a dextran-coated iron oxide NPs formulation formerly approved for clinical MRI, and to other iron-oxide loaded nanosystems 53,96, thus suggesting similar relaxometric properties for these magnetic systems.

A decreasing tendency was observed at 21.5 MHz (Table 1, Figure SM4) for both r_1_ and r_2_ relaxivities with the enhancement of magnetite content inside the capsule, with r_2_ values displaying a sharper decay. A similar trend has been previously reported for magnetic polymer micro- and submicron capsules 88, 97, where it was ascribed to the effect of clustering and inhomogeneous spatial distribution of the embedded MNPs within the capsules on the dipolar magnetic energy and on the water protons/MNP interaction. Interestingly, despite these r_1_ and r_2_ decays, their ratio (r_2_/r_1_) remained almost the same with increasing loading cycles up to 2 (samples S, C_1_S and C_2_S). Meanwhile, with the more drastic increase of magnetite content inside the capsule, as for the C_6_S sample, the possibility of MNP aggregation raised, leading to a significant r_2_/r_1_ decrease (see Table 1).

As far as the proposed contrast agent is capsule-based and the amount of MNPs inside these capsules differed depending on the sample structure, it was useful to compare also the relaxivities of the samples S and C_6_S normalizing them to the capsules' concentration (indicated as r_1caps_ and r_2caps_) instead of concentration of Fe(III) ions. Such comparison demonstrated no drastic difference between the obtained r_1caps_ values (9.3 vs 9.9 (pM**×**s)^-1^ for the S and C_6_S samples, respectively). However, the transverse relaxivity r_2caps_ for the sample S was still higher than for the sample C_6_S (43 vs 26 (pM**×**s)^‑1^, respectively).

**Figure 5:**
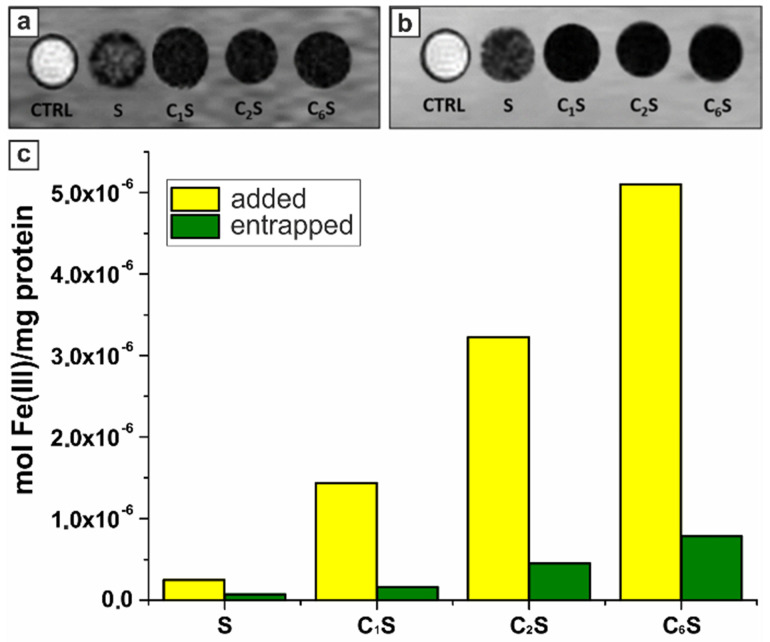
T_1_w (a) and T_2_w (b) magnetic resonance images acquired at 7 T of glass capillaries containing RAW 264.7 cells incubated for 2 hours in the absence (control cells, CTRL) and in the presence of magnetic polyelectrolyte capsules (samples S, C_1_S, C_2_S, C_6_S). (c) Histogram representing the amount of iron added to (yellow columns) and entrapped by (green columns) RAW 264.7 cells after the 2-hours incubation with samples S, C_1_S, C_2_S, and C_6_S represented as moles of iron per 1 mg of cellular proteins; the calculations were based on ICP-MS analysis of Fe(III) content and spectrophotometric evaluation of proteins by means of Bradford assay.

Before carrying out the *in vivo* study, the possibility to generate a sufficient MRI contrast was assessed for the RAW 264.7 cells labeled with the synthesized magnetic polyelectrolyte capsules. This murine phagocytic cell line was used as an immune cell model simulating capsules' uptake 98. T_1_ and T_2_-weighted MR images were acquired at 7 T on cellular pellets obtained upon 2‑hours incubation with MNP-doped capsules (samples S, C_1_S, C_2_S, C_6_S). In order to study the effect of the sample structure, the concentration of capsules added to cells was set at the same level for all the samples, while the total amount of incubated iron differed. The resulting T_1_- and T_2_-weighted MR images are presented in Figure 5 (a, b).

The MNP-doped polyelectrolyte capsules demonstrated the ability to be entrapped by macrophages generating sufficient MRI contrast in both T_1_ and T_2_ weighted images. The R_1obs_ and R_2obs_ relaxation rates were not measurable for the samples C_1_S, C_2_S and C_6_S due to the extremely high Fe(III) content. High iron concentration resulted in a strong T_2_ effect, affecting also T_1_w contrast as decreasing T_1_ signal intensity. While for the sample S, where the magnetite content was significantly lower, the T_1_ and T_2_ signal was much higher than for CnS ones. Thus, the r_1_ and r_2_ relaxivities (at 7 T and normalized to Fe(III) ions) were calculated just for the cells incubated with sample S and corresponded to 11.5 and 711.5 mM^-1^s^-1^, respectively. It should be noted that R_1obs_ and R_2obs_ values were more than 100 and 5 times higher than the relaxation rates measured for the control RAW 264.7 cells (incubated without capsules), respectively (29.6 vs 0.3 s^-1^ for R_1obs_; 92.7 vs 20.6 for R_2obs_). The relaxivities of the magnetic polyelectrolyte capsules acquired at 7 T are provided in Table SM1 (Supplementary Material).

The amount of iron entrapped by cells after their incubation with capsules was evaluated by ICP-MS. The mean Fe(III) content per mg of cellular protein for each sample was then calculated based on these data and presented in Figure 5c. These results demonstrated a good labeling ability *in vitro* rendering the proposed capsules-based contrast agent promising for *in vivo* MRI application. According to spectrophotometric evaluation of proteins with a Bradford assay, 1 mg of proteins was equal to 10^6^ RAW cells.

High amount of iron was entrapped also when the MNP-doped capsules were incubated with adenocarcinoma TS/A cells simulating the process of capsules' uptake in targeted tumor tissues (see Figure SM5 in SM). The drastic changes in MR properties of these tumor cells resulting from the internalization of the capsules were noted as well (Figure SM5 in SM). Accordingly, one might expect to observe the steep decrease of MR signal in tumor as soon as the proposed theranostic system is successfully delivered there.

**Figure 6:**
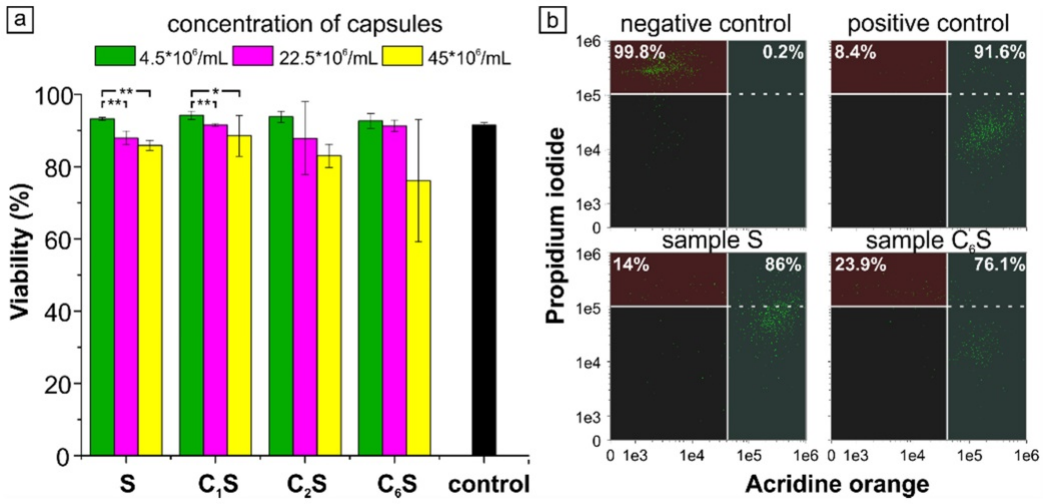
(a) Cytotoxicity of magnetic polyelectrolyte capsules S, C_1_S, C_2_S and C_6_S. The statistically significant differences calculated by ANOVA-test are represented as * (p<0.05) and ** (p<0.01). (b) Flow cytometry scatter plot for Acridine orange/Propidium Iodide double stained RAW 264.7 cells after capsules' uptake. Negative control represents cells without capsules after induced necrotic death; positive control represents cells without any additional treatment.

Cytotoxicity of the obtained capsules was also determined prior to the start of *in vivo* experiments. For this purpose, MNP-doped capsules of various concentrations were incubated with RAW 264.7 cells. For cytotoxicity assay, the largest number of capsules added to a dish of cells was 9x10^7^ (45x10^6^ capsules/mL of medium), as it corresponded to the number of capsules further administered to an average mouse (weighing 20 g) *in vivo*. The results are presented in Figure 6.

The study of macrophages viability under the 2-hour incubation with magnetic polyelectrolyte capsules of various structure performed using double AO-PI staining demonstrated a good cytocompatibility of the samples added even at the highest concentration of 450 capsules per cell (9x10^7^ capsules per dish). Although cell viability slightly varied with the capsules' structure and concentration, it was no less than 76% for the C_6_S sample (Figure 6, a). For a better illustration, a scatter plots showing the distribution of double stained AO-PI RAW 264.7 cells for the highest concentration of S and C_6_S samples were provided (Figure 6 b). As far as the viability was higher than 70% for all the samples, the carriers were considered non-cytotoxic in accordance with ISO 10993-5:2009 99. According to ANOVA-test, the change in capsule's structure for the same capsule concentration did not lead to any statistically significant differences in RAW 264.7 cell viability. The change in carrier concentration caused statistically significant difference only for S and C_1_S samples, while for the others the differences were not statistically significant even if the average values were noticeably lower.

Based on the data obtained, the capsules with MNPs incorporated only into their shells (sample S) were selected as the best T_2_ contrast agent in terms of MRI visualization properties *in vitro*. Meanwhile, the capsules containing the highest amount of magnetite (MNPs in the shell and inner volume of the capsules, sample C_6_S) revealed as the optimal system concerning the highest sensitivity to a magnetic field and possibility to generate a sufficient negative MRI contrast upon the cellular internalization. At the same time, both samples applied even at a high concentration did not cause a prominent cytotoxic effect. By this means, the samples S and C_6_S were chosen for the following *in vivo* assessment.

### Magnetic polyelectrolyte capsules in grafted breast tumor mouse model

The possibility to deliver the obtained magnetic polyelectrolyte capsules to a tumor was then studied by means of *in vivo* MRI. To be effective, the proposed theranostic system needs to provide a good balance between MR contrast and magnetic navigation properties when applied *in vivo* as we aimed at successful magnetic targeting realization.

TS/A cells were used to form a grafted tumor model in mice. It is well known that high permeability of the tumor vasculature compared to normal tissue allows large molecules and small particles to enter the tumor interstitial space and the compromised lymphatic filtration allows them to stay there (EPR effect) 100. As far as the permeabilized vasculature varies from 200 to 800 nm, such passive targeting effect is expected to take place for the proposed delivery system as well. That should result in intratumor accumulation of the capsules and, thus, in MR contrast enhancement. Assuming this possible effect, the control experiments on MNP-doped capsules' injection not followed by external magnet application (so-called “no magnet”) were performed to estimate the real effect of magnetic targeting.

**Figure 7:**
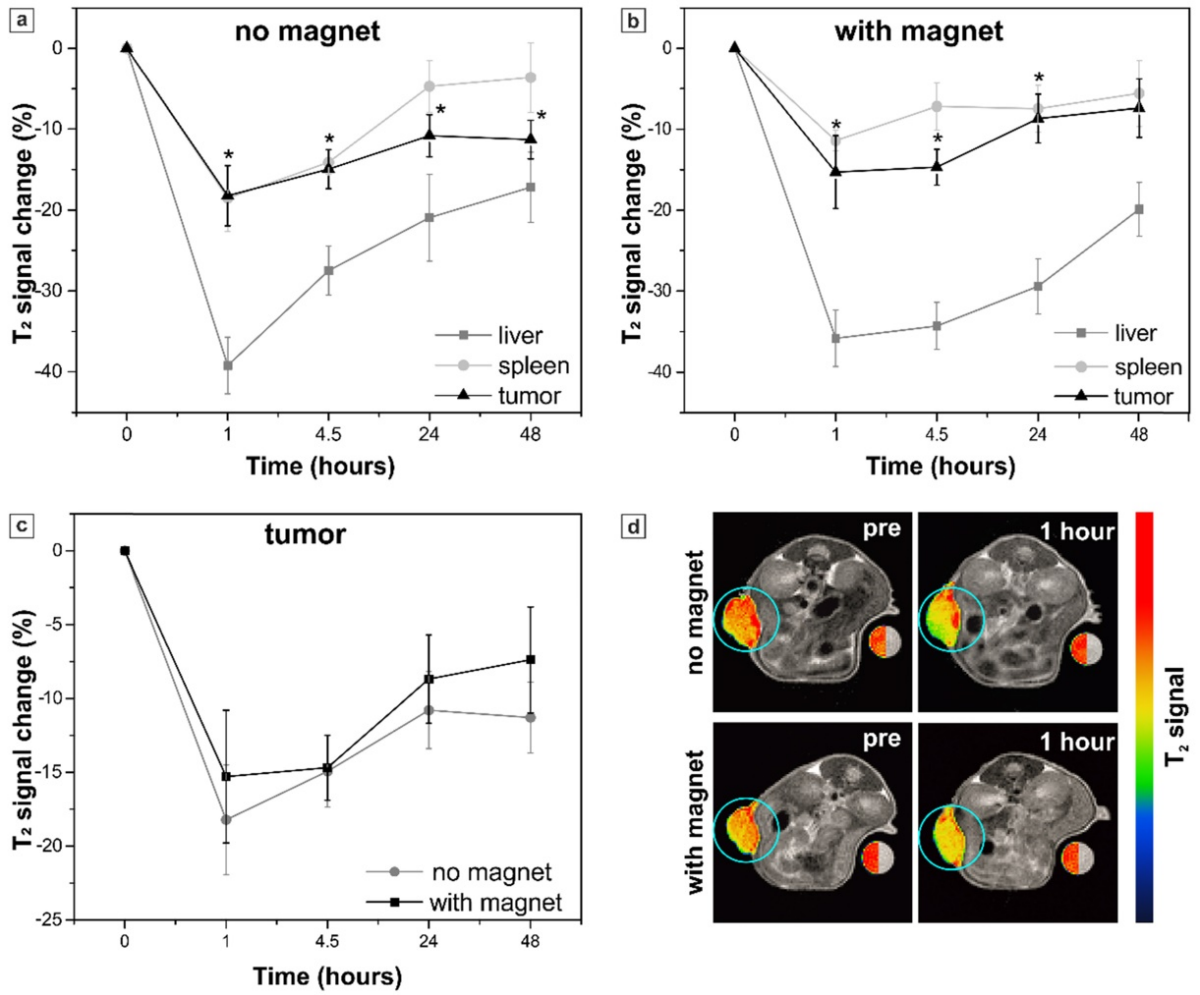
Sample S. Percent of T_2_ signal change in the organs of interest (tumor, liver and spleen) at different time points resulting from the systemic injection of sample S: (a) without and (b) with the application of the external magnet to the tumor, an asterisk (*) indicates significant differences in T_2_ signal change in tumor in comparison to pre-injection images; (c) comparison of T_2_ signal change in the mice tumors with and without magnet application. The values correspond to the “mean ± SE”, n=6 per each group (no magnet/ with magnet). (d) Representative T_2_-weighted axial MR images of mice acquired before (pre) and 1 hour after the injection. Tumors are marked with blue circles and presented in pseudo colors; a water-containing reference tube is presented in both, original grayscale and pseudo colors.

The sample S was tested first as providing better T_2_ contrast properties. 4 nmol Fe(III)/g (4.5x10^6^ capsules/g body weight) were administered to mice (n=6) by intravenous injection. The accumulation in tumor, liver and spleen tissues was assessed 1, 4.5, 24 and 48 h after the injection by acquiring MR images at 1 T and calculating T_2_ contrast. The contrast was expressed as the “percent of T_2_ signal change” representing the percentage change in the mean signal intensity of the organ of interest in comparison to pre-injection images (see Material and Methods). The results are shown in Figure 7 (a).

Injection of sample S provided generation of a marked MRI contrast* in vivo*. The maximum of T_2_ signal decrease in tumor (-18 ± 4 %) was registered 1 hour after the injection, meaning that the amount of the MNP-doped capsules in tumor was maximal at this time point. The systemic clearance of not internalized capsules led to a sufficient decrease in T_2_ signal intensity in liver and spleen as well (-39 ± 4 % and -19 ± 4 %, respectively).

In order to improve the tumor accumulation of the proposed submicron capsule-based MR probe, magnetic targeting was applied then. To accomplish this, a permanent magnet with a concentrator (0.5 T) was placed on the tumor right before the injection of capsules (Figure 1, Materials and Methods) and kept applied for 1 hour further. MR images were acquired again at 1 T and T_2_ contrast was measured. The obtained results are presented in Figure 7 (b). The comparison of the mean T_2_ signal change in the mice tumors with and without magnet application is presented in Figure 7 (c, d).

The maximum of T_2_ signal decrease in tumor was again reached 1 h after the injection. However, no significant difference was observed between the groups of mice measured with and without the external magnet application, neither 1 hour after the injection (‑15 ± 5 % and -18 ± 4 %, respectively), nor at the later time points (Figure 7c). Quantification of the T_2_ signal in liver and spleen did not show any significant impact of the magnet application as well. Meanwhile, the axial MR images of mice (Figure 7d) demonstrated the absence of signal artefacts in tumor after the application of the magnet that presumably resulted from a more homogeneous distribution of the magnetic particles. By this means, the exposure to a magnetic field may allow the overcoming of the impaired drugs/nanosystems' distribution often observed in tumors due to disordered vasculature, hypoxic microenvironments and elevated interstitial fluid pressure 40,101.

As mentioned above, the MR contrast enhancement in the tumor indicates a successful targeting of the contrast agent. Thus, polyelectrolyte capsules with MNPs incorporated into their shells (sample S), which played a role of such agent here providing almost 20% T_2_ signal change, appeared to be a promising delivery system. Once co-loaded with an anticancer drug, these capsules may contribute to its accumulation in tumor tissue. Furthermore, gradual fading of T_2_ contrast within 48 hours in both spleen (approaching the pre-injection values) and liver (recovering to half of the pre values) took place in our study (Figure 7 a, b). That pointed at a relatively short period of capsules' elimination allowing for repeated administrations in anticancer therapy. Realization of magnetic targeting of polyelectrolyte capsules to the tumor, which has failed for sample S due to its low sensitivity to external magnetic field, might enhance the amount of a delivered drug as well. However, the more sensitive magnetic system should be used for this purpose.

**Figure 8:**
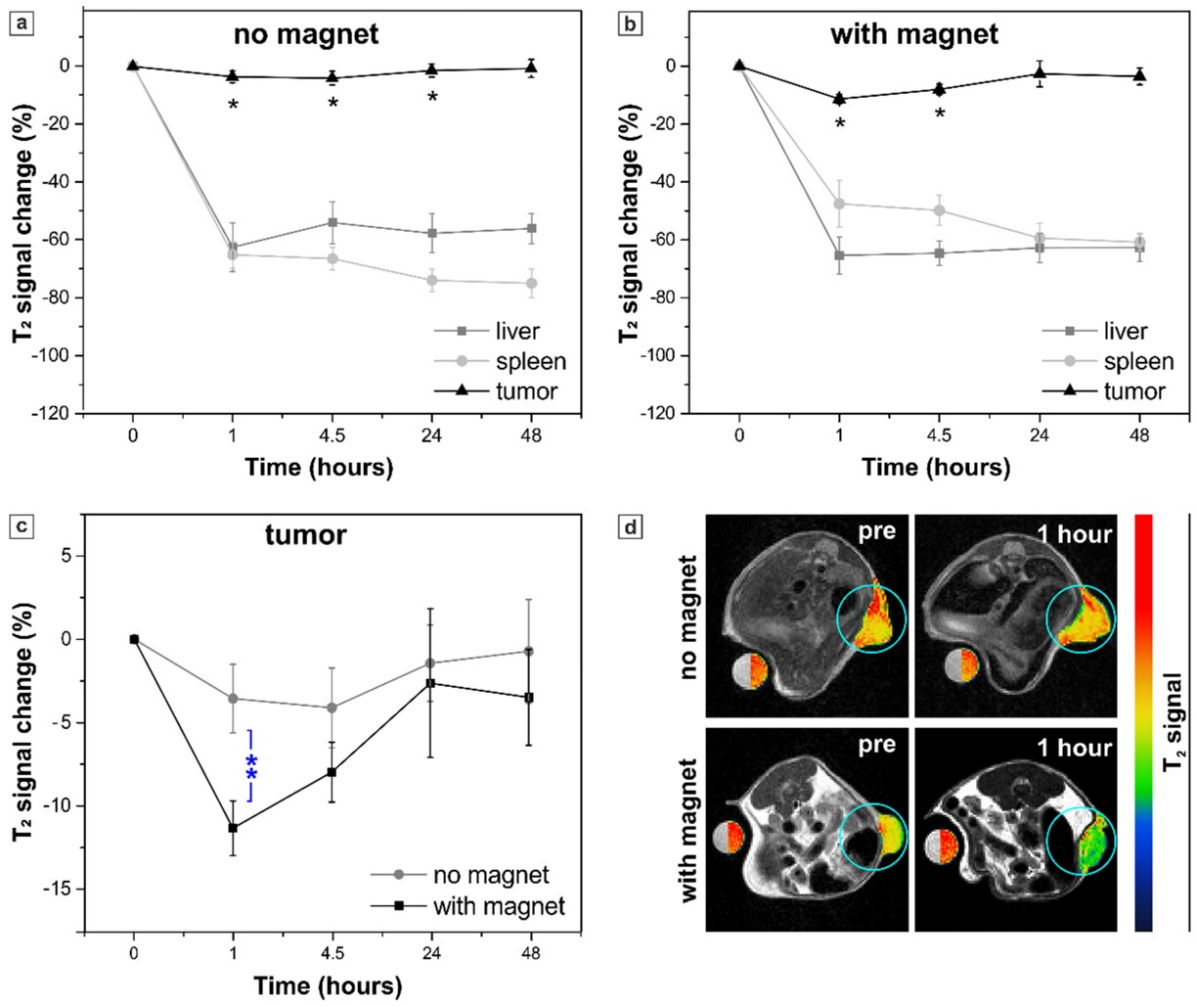
Sample C_6_S. Percent of T_2_ signal change in the organs of interest (tumor, liver and spleen) at different time points resulting from the systemic injection of sample C_6_S: (a) without and (b) with the application of the external magnet to the tumor, an asterisk (*) indicates significant differences in T_2_ signal change in tumor in comparison to pre-injection images; (c) comparison of T_2_ signal change in the mice tumors with and without magnet application, two asterisks (**) indicate significant difference (*p* < 0.01) between these two groups. The values correspond to the “mean ± SE”, n=4 per each group (no magnet/ with magnet). (d) Representative T_2_-weighted axial MR images of mice acquired before (pre) and 1 hour after the injection. Tumors are marked with blue circles and presented in pseudo colors; a water-containing reference tube is presented in both, original grayscale and pseudo colors.

Thus, in the second set of experiments, *in vivo* MRI was carried out in mice receiving sample C_6_S as it demonstrated the highest sensitivity to a magnetic field *in vitro*. Here, 147 nmol Fe(III)/g were intravenously administered to mice (n=4) maintaining the same capsules' concentration used for the sample S (4.5x10^6^ capsules/g body weight). The T_2_ contrast in tumor, liver and spleen tissues was assessed again 1, 4.5, 24 and 48 h after the injection by acquiring MR images at 1 T and the resulting data are shown in Figure 8.

Injection of the sample C_6_S resulted in a significant T_2_ signal decrease (in comparison to pre injection values) in tumors of both mice groups, exposed or not to the external magnetic field (Figure 8 a, b). However, no enhancement in MRI contrast was observed in tumor if compared to the treatment with sample S, while the mean T_2_ signal in liver and spleen decreased drastically. Such an effect is caused by the dense packing of MNPs in the inner volume of the C_6_S capsules. As described above, a high amount of magnetite inside the capsule negatively affects the T_2_ contrast properties of the sample (Table 1). Enhanced local concentration of MNPs promotes a strong “quenching” effect on the observed relaxivity of intact capsules 46,102. That resulted in a less accentuated T_2_ signal decrease in tumor for the sample C_6_S as compared with sample S. Meanwhile, upon the degradation of MNP-doped capsules, liberation of magnetite particles together with the enhancement of interparticle distance take place causing a sufficient change in MR contrast of the reticuloendothelial system (RES)-associated organs 46. Here, it resulted in such a drastic decrease of T_2_ signal in liver and spleen after the injection of sample C_6_S in comparison to sample S, especially 48 hours post injection. Specifically, in liver the mean T_2_ signal change values obtained at this time point for sample C_6_S exceeded the ones obtained for sample S by 3 times. In spleen a more than 10-times higher T_2_ signal decrease and thus, T_2_ contrast enhancement, was registered 48 hours after the injection of sample C_6_S (compared again to treatment with sample S).

Nevertheless, the main effect associated with the injection of highly loaded polyelectrolyte/magnetite capsules (sample C_6_S) occurs when comparing their injection with and without an external magnetic field application (Figure 8 c, d). The capsules have demonstrated a good magnetic sensitivity *in vivo* providing a significantly stronger (*p* < 0.01) T_2_ contrast 1 hour after the injection in tumor exposed to magnet (-12 ± 2 % vs -4 ± 2 % T_2_ signal change for mice treated and not treated with magnet, respectively). At the same time, significantly lower (*p* < 0.05) T_2_ contrast was observed in spleen of the treated with magnet mice (T_2_ signal change was -43 ± 7 %, while for untreated mice it corresponded to -65 ± 5 %). Both facts pointed at successful magnetically guided targeting of the sample C_6_S to tumor.

**Figure 9:**
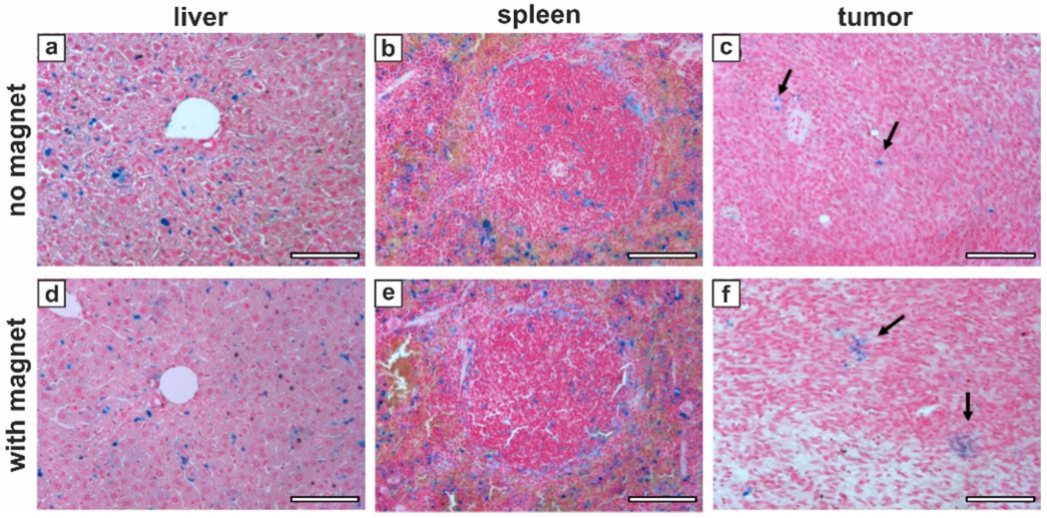
Perls' staining of representative mice tissues 48 hours after the injection of sample C_6_S without (a-c) and with (d-f) the application of an external magnet: (a, d) - liver; (b, e) - spleen; (c, f) - tumor. Black arrows indicate iron deposits in tumor. Scale bars, 100 µm.

In order to validate this effect of external magnet application and to verify that MNP-doped capsules were indeed delivered to tumor tissue, mice were sacrificed after the last MRI acquisition (48 hours after the injection of sample C_6_S) to carry out histological analysis of their liver, spleen and tumor. The Perls' (Prussian Blue) staining protocol was used to assess localization of the capsules and iron deposits in tissues. The results are presented in Figure 9.

The Perls' staining positivity of histological sections is shown for all the investigated organs indicating the presence of iron deposits 48 hours after the sample injection (Figure 9), that well matched the MRI results (Figure 8 a-c). No marked difference between the groups with and without magnet application was found for liver (Figure 9 a, d) and spleen (Figure 9 b, e) tissues, where the iron was located almost exclusively in macrophages. In tumor, instead, even if the amount of residual contrast agent by this time was limited for both groups, the arrangement of MNP-doped capsules/iron deposits differed significantly (Figure 9 c, f). That clearly shows that an hour-long application of external magnet to tumor following the intravenous injection of the magnetically sensitive capsules resulted in a denser and clustered positioning of the capsules in tumor tissue, as the formed aggregates remained there even 47 hours after removing the magnetic field.

By this means, the obtained results indicated a successful realization of the magnetic targeting using the proposed MNP-doped polyelectrolyte capsules that might guarantee the delivery of a fairly higher amount (at least 3 times, relying on T_2_ contrast observed) of a co-loaded drug into the tumor under external magnetic field. Such enhanced targeted delivery of polyelectrolyte capsules offers great perspectives for drug delivery improvement.

## Conclusions

In summary, the current study highlighted the strategy of magnetic biodegradable polyelectrolyte carriers' design to develop an efficient delivery system combining the externally controlled navigation and MRI visualization properties. The effect of changing the structure of polyelectrolyte/magnetite submicron capsules and the amount of incorporated magnetite has been studied systematically moving from *in vitro* to *in vivo*. Incorporation of magnetite nanoparticles into the inner volume of capsules in addition to their shell labeling significantly improved the magnetic targeting ability. Intravenous injection of the most highly loaded sample containing 2.94 μmol Fe(III) to mice with a breast cancer model under external magnet application resulted in a three-fold enhancement in T_2_ MRI contrast in tumor (compared to the tumor untreated with magnet) together with a pronounced contrast lowering in spleen. Such findings rendered the proposed magnetic capsules effective in terms of both external magnetic field-guided targeting of tumors and MRI monitoring. Summation of these properties of the polyelectrolyte capsules with biocompatibility and the ability of co-loading with an anticancer drug holds the prospect for the effective theranostic platform development aiming at improved anticancer therapy. Further research should be aimed at the application of external physical stimuli (light, ultrasound or alternating magnetic field) providing controlled *in-situ* drug release or synergetic therapeutic effect in tumor.

## Supplementary Material

Supplementary figures and tables.

## Figures and Tables

**Table 1:** Structure and characteristics of the magnetic polyelectrolyte capsules at 0.5 Т and 25°C

Sample	Structure	[Fe^3+^] mM	r_1_ (mM×s)^-1^	r_2_ (mM×s)^-1^	r_2_/r_1_
*S*	(PA/DS/PA/**MNPs**/PA/DS)	0.43	19.7	92.1	4.7
*C_1_S*	(**MNPs**)/(PA/DS/PA/**MNPs**/PA/DS)	4.4	17.8	79.3	4.5
*C_2_S*	(**MNPs**)_2_/(PA/DS/PA/**MNPs**/PA/DS)	8.8	5.3	23.4	4.4
*C_6_S*	(**MNPs**)_6_/(PA/DS/PA/**MNPs**/PA/DS)	15.8	0.6	1.5	2.7
