# Supplementary material for: Biodegradable polyelectrolyte/magnetite capsules for MR imaging and magnetic targeting of tumors
Source: Nanotheranostics. 2021 Apr 2;5(3):362–77. doi: 10.7150/ntno.59458 (PMC8040826; doi:10.7150/ntno.59458)
Supplement: Supplementary file 1 — Supplementary figures and tables. [file ntnov05p0362s1.pdf]

SUPPLEMENTARY MATERIAL

Biodegradable polyelectrolyte/magnetite capsules for MR imaging and magnetic targeting of tumors

Yulia Svenskaya[1,,^], Francesca Garello[2,^], Ekaterina Lengert[1], Anastasiia Kozlova[3], Valeria Bitonto[2], Roman Verkhovskii[3], Maria Rosaria Ruggiero[2], Sergey German[4,5], Dmitry Gorin[5], Enzo Terreno[2,*]*

[1] Remote Controlled Systems for Theranostics laboratory, Research and Educational Institute of Nanostructures and Biosystems, Saratov State University, 410012 Saratov, Russia

[2] Molecular and Preclinical Imaging Centres, Department of Molecular Biotechnology and Health Sciences, University of Torino, 10126 Torino, Italy

[3] Biomedical Photoacoustics Laboratory, Saratov State University, 410012 Saratov, Russia

[4] Laboratory of Optics and Spectroscopy of Nanoobjects, Institute of Spectroscopy of the RAS, Troitsk 108840, Russia

[5] Center of Photonics and Quantum Materials, Skolkovo Institute of Science and Technology, 143026 Moscow, Russia

^ Yu. Svenskaya and F. Garello contributed equally to this work

Corresponding authors: Yulia Svenskaya, e-mail: svenskaya@info.sgu.ru; Enzo Terreno, e-mail: enzo.terreno@unito.it

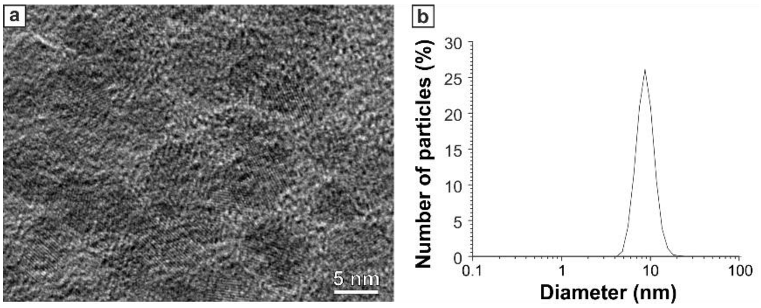

Figure SM1. (a) TEM image of colloidal dispersion of magnetite nanoparticles. (b) Distribution of magnetite nanoparticle diameter measured by DLS.

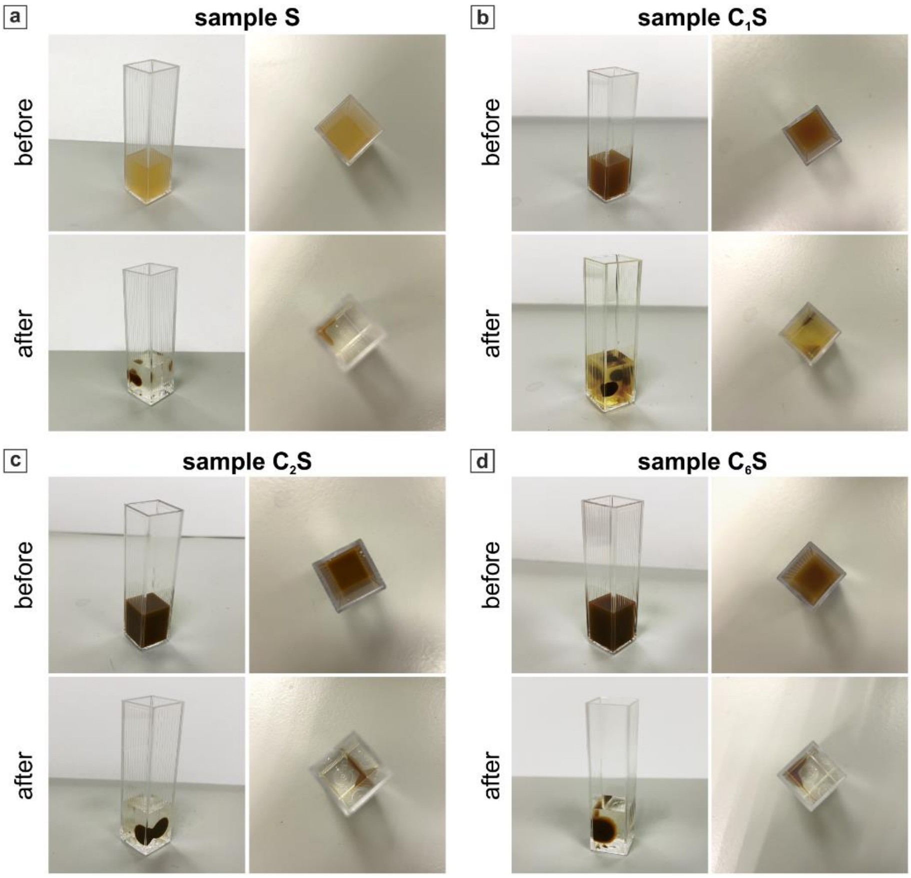

Figure SM2. Photographs of the capsule suspensions in cuvette before and after the application of the permanent magnet with a concentrator (0.5 T).

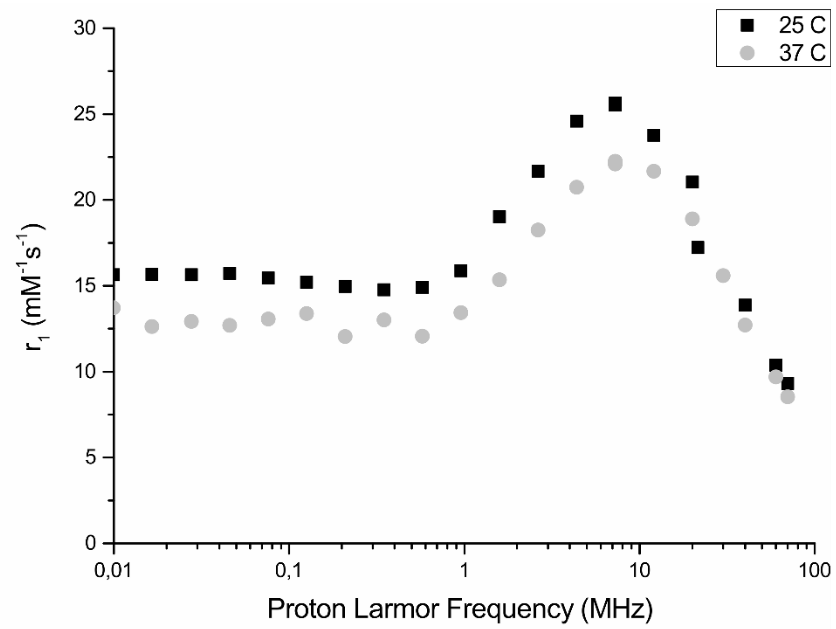

Figure SM3. ^{1}H Nuclear Magnetic Relaxation Dispersion (NMRD) profiles of magnetic polyelectrolyte capsules (sample S) acquired at 25 (black squares) and 37 °C (grey circles).

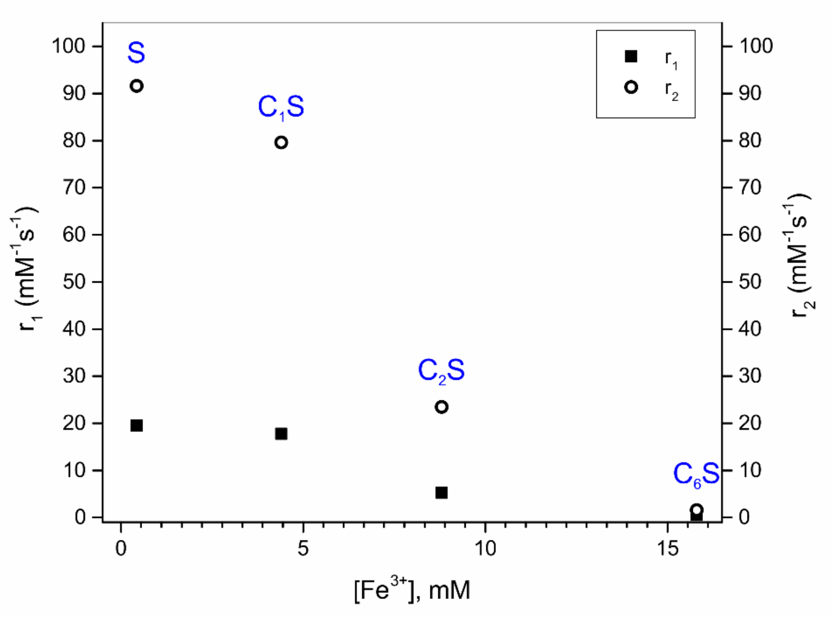

Figure SM4. Dependence of the longitudinal (r_1) and transverse (r_2) relaxivities on the amount of iron in the sample at 0.5 T and 25 °C.

Table SM1. Characteristics of magnetic polyelectrolyte capsules at 7 T and 25 °C.

Sample	Structure	r_1, (mM×s)$^{-1}$	r_2, (mM×s)$^{-1}$	r_2/r_1
C_1S	(MNPs)/(PA/DS/PA/MNPs/PA/DS)	0.6	90.7	147.5
C_6S	$(MNPs)_6$/(PA/DS/PA/MNPs/PA/DS)	0.2	36.5	197.0

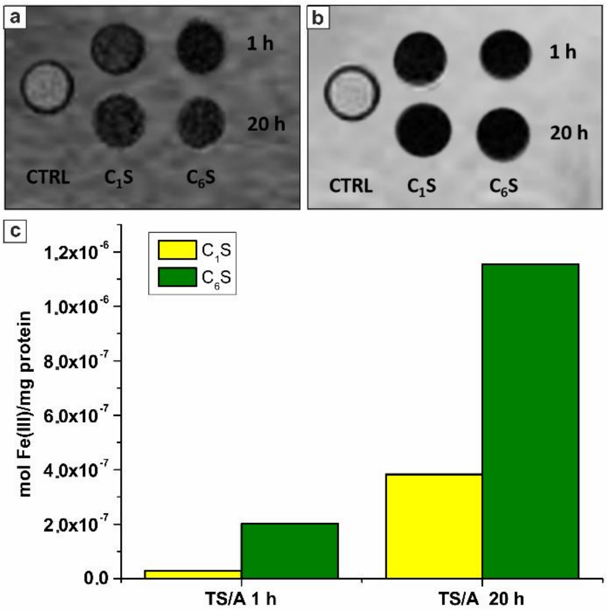

Figure SM5. T_{1w} (a) and T_{2w} (b) magnetic resonance images acquired at 7 T of glass capillaries containing TS/A cells incubated for 1 and 20 hours in the absence (control cells, CTRL) and in the

presence of MNP-doped capsules C_1S and C_6S; (c) amount of iron (in mol) per 1 mg of cellular proteins calculated in TS/A cells following the incubation with sample C_1S or C_6S. 1 mg of proteins is equal to $2.5x10^6$ TS/A cells [di Gregorio E, Ferrauto G, Gianolio E, Aime S. Gd loading by hypotonic swelling: an efficient and safe route for cellular labeling. Contrast Media Mol. Imaging, 2013; 8: 475-486].
